# Chatting with the neighbors: crosstalk between Rho-kinase (ROCK) and other signaling pathways for treatment of neurological disorders

**DOI:** 10.3389/fnins.2015.00198

**Published:** 2015-06-02

**Authors:** Niko Hensel, Sebastian Rademacher, Peter Claus

**Affiliations:** ^1^Hannover Medical School, Institute of NeuroanatomyHannover, Germany; ^2^Niedersachsen Research Network on NeuroinfectiologyHannover, Germany; ^3^Center for Systems NeuroscienceHannover, Germany

**Keywords:** spinal muscular atrophy (SMA), amyotrophic lateral sclerosis (ALS), huntington’s disease (HD), parkinson’s disease (PD), alzheimer disease (AD), multiple sclerosis (MS), neuroinflammation, microglia

## Abstract

ROCK inhibition has been largely applied as a strategy to treat neurodegenerative diseases (NDDs) and promising results have been obtained in the recent years. However, the underlying molecular and cellular mechanisms are not fully understood and different models have been proposed for neurodegenerative disorders. Here, we aim to review the current knowledge obtained for NDDs identifying common mechanisms as well as disease-specific models. In addition to the role of ROCK in different cell types such as neurons and microglia, we focus on the molecular signaling-pathways which mediate the beneficial effects of ROCK. Besides canonical ROCK signaling, modulation of neighboring pathways by non-canonical ROCK-crosstalk is a recurrent pattern in many NDD-model systems and has been suggested to mediate beneficial effects of ROCK-inhibition.

## Introduction

**Figure 1:**
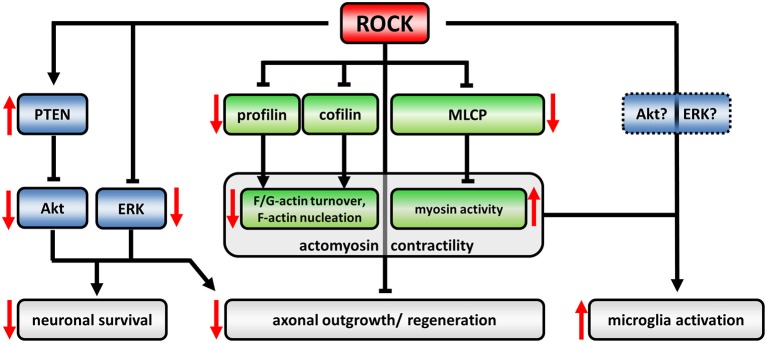
**The ROCK-pathway in neurons and microglia**. The regulatory network downstream of ROCK is defined by activating (arrows) or inactivating (blunted arrows) interactions. Induction of ROCK-activity leads to an up- or down-regulation of downstream targets, respectively (effects of upregulated ROCK-activity represented by red arrows). ROCK controls actomyosin contractility (canonical signaling, green) as well as Akt- and ERK-activity (non-canonical crosstalk, blue). ROCK-dependent profilin phosphorylation reduces its recycling capacity for globular (G)-actin leading to decreased turnover between filamentous (F) and G-actin. Cofilin-phosphorylation inactivates its F-actin severing activity resulting in less F-actin nucleation. Phosphorylation and inactivation of myosin light chain phosphatase (MLCP) induces myosin activity facilitating retrograde flow of F-actin. Together, those changes lead to a collapsing growth cone and inhibit axonal regeneration processes (reviewed in Lowery and Van Vactor, 2009; Gomez and Letourneau, 2014). ROCK phosphorylates and activates PTEN which in turn inactivates Akt. Similarly, ERK-activity is negatively regulated by ROCK. Neurotrophic Akt- and ERK-pathways are well described agonists of neuronal survival. However, also neuronal morphology is targeted by Akt/ERK signaling thereby influencing neuronal regeneration processes. The role of ROCK activity in microglia-activation is less understood. However, cytoskeletal alterations in ROCK-inhibitor treated cells implicate an involvement of canonical signaling, while ROCK-crosstalk has not been evaluated in microglia yet.

An important signaling hub for the regulation of the actin cytoskeleton and myosin-based contractility is the Rho-kinase (ROCK). Signaling events downstream of ROCK are responsible for key neuronal processes in axonogenesis, growth cone dynamics, and stability of synapses. ROCK has a number of downstream targets including profilins and cofilin (via LIM kinase) as actin-binding proteins as well as myosin light chain phosphatase (MLCP) (Figure 1). Upstream of ROCK, Rho-GTP or phosphatidylinositol-4,5-bisphosphate (PIP_2_), and phosphatidylinositol-(3,4,5)-trisphosphate (PIP_3_) activate ROCK (reviewed in Tonges et al., 2011). ROCK-activity mainly acts detrimental on clinically relevant outcomes in neurodegenerative diseases (NDDs). This has been elucidated in the recent years by a series of *in vivo* animal studies: Expression of dominant negative ROCK-isoforms or application of small molecule inhibitors in rodent-models of several NDDs or diseases with a neurodegenerative component impressively ameliorated disease-phenotypes in many studies. Thereby, small molecule inhibitors are of special interest because of their potential translational use in the clinics compared to genetic approaches. Among those, Y27632 has been extensively used in basic science. However, since now there is only a single ROCK-inhibitor, Fasudil, which obtained drug approval by Japanese authorities for ischemic stroke treatment (Chen et al., 2013a). Together with the promising results in NDD animal models this hints for broad neuroprotective effects of ROCK-inhibition. However, distinct mechanisms are attributable for that.

**Figure 2:**
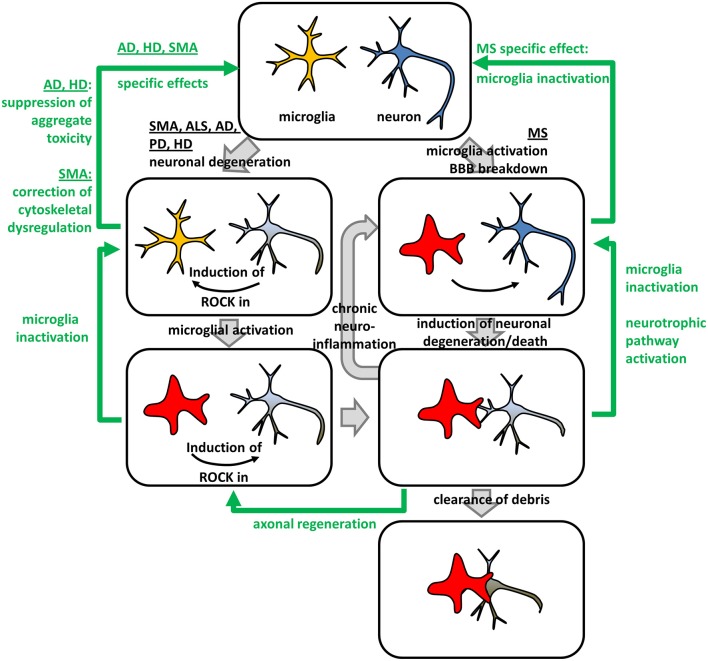
**Beneficial effects of ROCK-inhibition in neurodegeneration – an integrated model**. Neurodegenerative and neuroinflammatory processes (gray arrows) in different NDDs and in multiple sclerosis (MS) can be reversed by ROCK-inhibition (green arrows). Neuronal degeneration in Spinal Muscular Atrophy (SMA), Amyotrophic Lateral Sclerosis (ALS), Alzheimer's Disease (AD), Parkinson's Disease (PD), and Huntington's Disease (HD) lead to a microglia-intrinsic ROCK-induction necessary for microglia-activation. Activated microglia form engulfing “gliapses” with degenerating neurons subsequently phagocytosing neuronal debris. Moreover, they secret factors, which activate ROCK in neurons, thereby inhibiting axonal regeneration. Activated microglia can enter a “vicious circle” of chronic neuroinflammation attacking healthy neurons leading to neuronal degeneration and death. In MS, chronic neuroinflammation might be induced by microglia-activation and Blood-Brain Barrier (BBB) breakdown. ROCK-inhibition interferes with different pathomechanistic alterations in NDDs (green arrows). Down-regulation of ROCK-hyper-activity in microglia leads to their inactivation, while neuron-intrinsic ROCK-inhibition triggers axonal regeneration via canonical ROCK-signaling controlling actomyosin contractility. Additionally, ROCK-inhibition activates neurotrophic pathway-signaling via non-canonical crosstalk leading to a neuronal rescue. Besides those general effects, ROCK-inhibition specifically interferes with disease-specific molecular pathomechanisms. In AD and HD, neuronal aggregate toxicity is attenuated by ROCK-inhibition, while cytoskeletal dysregulations involving profilin represent the underlying mechanism in SMA.

Here, we aim to review the current literature of ROCK-inhibition bringing together different fields of NDDs, thereby identifying common mechanisms including crosstalk with other downstream pathways as well as disease specific effects. Anticipating this, enhanced ROCK-activities have been reported in many *in vitro* and *in vivo* NDD models. Thus, ROCK inhibition might at least in part exert its beneficial effects on NDDs by rescuing pathomechanistic changes. Thereby, two dimensions of elevated ROCK-activity have to be taken into account: (i) The pathway dimension, and (ii) the cell-type dimension. (i) Canonical ROCK signaling has been extensively studied and associated with the control of actomyosin contractility via phosphorylation of downstream actin-binding proteins such as cofilin or profilin as well as myosin-binding proteins such as myosin light chain phosphatase (reviewed in Gomez and Letourneau, 2014) (Figure 1). In neuronal cells, globally enhanced ROCK-activity leads to growth cone collapse indicating a detrimental role in regenerative, outgrowth related processes (Lowery and Van Vactor, 2009). Despite that, ROCK negatively controls neurotrophic pathway-signaling, which we will term “non-canonical ROCK-crosstalk”. Those pathways involve PTEN, Akt, and ERK acting upstream of neuronal survival (Lingor et al., 2008; Takata et al., 2013; Hensel et al., 2014). Thus, enhanced neuronal ROCK activity in NDDs might inhibit regeneration as well as survival (Figure 1). However, the mechanisms of the crosstalk still lack a detailed molecular description.

(ii) In microglia, ROCK-activity is critically involved in neurodegeneration. Despite microglia, other inflammatory cells are likely involved in neurodegeneration. However, in the context of ROCK-inhibition in NDDs microglia have been most extensively studied. Microglia, also termed “macrophages of the nervous system”, have ambiguous roles in NDDs dependent on their activation state (reviewed in Tang and Le, 2015): Activated M1 microglia represent an early reaction to neuronal insults ensuring an inflammatory and neurotoxic environment. However, neurotoxicity might affect healthy neurons inducing a vicious cycle with chronic neuroinflammation (Tang and Le, 2015). In contrast, alternatively activated or deactivated M2-microglia are anti-inflammatory and support tissue and extracellular matrix repair (Tang and Le, 2015). Interestingly, ROCK activity is needed to maintain M1-phenotype as Fasudil-mediated ROCK-inhibition shifts M1-microglia to the M2-state (Zhang et al., 2013). Moreover, activation of the microglial ROCK-axis was associated with cytoskeletal changes and enhanced migration indicating involvement of canonical ROCK-signaling (Bernhart et al., 2010). Additionally, superoxide production was increased (Moon et al., 2013). In turn, microglia express repellents such as chondroitin sulfate proteoglycans (CSPG) which activate ROCK in neurons, thereby inhibiting axonal regeneration (Monnier et al., 2003; Koch et al., 2014). Thus, the benefits of ROCK-inhibition rely on the manipulation of cellular and molecular (patho-) mechanisms which are at least in part shared by all NDDs. Those mechanisms can be categorized into (i) support of axonal regeneration, (ii) support of neuronal survival, and (iii) inhibition of detrimental neuroinflammation. Moreover, we and others showed that ROCK-inhibition beneficially interferes with pathological molecular mechanisms specific for a single NDD (Figure 2).

## Parkinson's disease (PD)

PD is characterized by a loss of dopaminergic neurons within the substantia nigra pars compacta leading to an imbalanced basal ganglia signaling. Clinically, this manifests in severe motor impairments such as tremor, rigor, bradykinesia, and postural instability. Although a number of environmental risk factors have been linked with some cases of sporadic PD, the vast majority still remains of unknown etiology (reviewed in Schiesling et al., 2008). Beside these idiopathic PD cases, several genes have been identified to be involved in familial Parkinsonism. Among those, alpha-synuclein was the first to be identified (Polymeropoulos et al., 1997). Together with autosomal dominant point mutations, gene multiplications of wild-type alpha-synuclein have been linked to an enhanced susceptibility for PD (Singleton et al., 2003; Nishioka et al., 2006). The latter argues for a pathological gain of function mechanism. Interestingly, alpha-synuclein is also a major component of Lewy bodies (LBs) (Spillantini et al., 1997). LBs are neuronal cytoplasmic protein aggregates found in surviving neurons *post mortem* and represent a central pathological hallmark of idiopathic PD (Gibb and Lees, 1989). This hints for alpha-synuclein as a common pathomechanistic internode in PD-etiology. Supporting this, toxin-induced dopaminergic neurodegeneration, classically employed as PD model, is dependent on alpha-synuclein abundance: Knock-out mice were resistant to 1-methyl-4-phenyl-1,2,3,6-tetrahydropyrine (MPTP)-induced toxicity of dopaminergic cells (Dauer et al., 2002). Importantly, mutant alpha-synuclein transduced neuronal cultures as well as MPTP-based *in vivo* models displayed a hyper-active ROCK-signaling axis, emphasizing ROCK-upregulation as a common mechanism of genetic and toxin-induced PD-models (Barcia et al., 2012; Villar-Cheda et al., 2012; Tonges et al., 2014b).

Such toxin-induced rodent models have also been extensively employed to study the impact of ROCK-inhibition on clinically relevant outcomes in PD (Barcia et al., 2012; Tonges et al., 2012, 2014b; Tatenhorst et al., 2014; Saal et al., 2015). The severity of dopaminergic neuron damage might thereby be critical for the success of ROCK inhibition: While severely lesioned 6-hydroxy dopamine (6-OHDA) animals did not respond to oral Fasudil-application with respect to dopaminergic neuron numbers or motor-phenotype (Tatenhorst et al., 2014), the less severe MPTP-mouse displayed a partial rescue of dopaminergic neuron numbers within the substantia nigra, a partial rescue of TH-fiber density as well as motor-behavior (Tonges et al., 2012). Moreover, an up-regulation of the activity of the pro-survival Akt-pathway was reported in response to co-application with MPP+, the toxic MPTP-metabolite, and Fasudil *in vitro* (Tonges et al., 2012). As the MPTP-model displays an axonal dying-back pathology preceding neuronal demise (Li et al., 2009a), the authors suggested a dual impact of ROCK-inhibition on (i) axonal regeneration supported by (ii) induction of pro-survival pathways. The first might thereby be mediated by canonical ROCK-signaling controlling actomyosin contractility at the tip of the regenerating axon while the latter might be dependent upon non-canonical ROCK-crosstalk partners such as Akt.

In general, such non-canonical crosstalk is not restricted to Akt, but seems to involve neurotrophic signaling more broadly: *In vitro* and *in vivo* results demonstrated increased activities of STAT3, ERK, and Akt – classical neurotrophic pathways – when treated with ROCK-inhibitor Y27632. Importantly, these crosstalk were dependent on neurotrophic pathway activation as they could only be detected under co-treatment with neurotrophic factors such as Ciliary Neurotrophic Factor (CNTF) or Fibroblast Growth Factor 1 and 2 (FGF1, FGF2) (Lingor et al., 2008; Lin et al., 2009; Hensel et al., 2014). Additionally, we could show that the crosstalk between ROCK and ERK is bi-directional which might have important implications for the pathological situation in NDDs in general: Enhanced neuronal ROCK-activities in NDDs might not only hamper axonal regeneration via changed acto-myosin contractility but also shift neurons toward pro-apoptotic signaling (Hensel et al., 2014).

Interestingly, MPTP-induced reduction of TH+-cell numbers were also partially rescued in primary neuron/glia cultures by application of the ROCK-inhibitor Y27632 while there was no effect on pure neuronal cultures. In contrast, Y27632 was able to rescue neurite lengths independent of microglia abundance (Borrajo et al., 2014). This points toward cell-type specific beneficial roles of ROCK inhibition in PD: Supporting axonal regeneration via neuron-intrinsic pathways as well as inhibition of dopaminergic neuron loss via microglia-dependent mechanisms. The importance of microglia as a target for ROCK-inhibition in PD has been further underlined as MPP+-treatment led to an enhanced ROCK-activity in glia-cells *in vitro* (Villar-Cheda et al., 2012). Accordingly, MPTP-treatment induced microglial activation leading to an increase in contacts between microglia and dopaminergic neurons *in vivo* (Barcia et al., 2012). Those contacts, displayed distinct morphological properties with F-actin clusters polarized toward the microglia-neuron contact side engulfing degenerating neurons, similar to the immunological synapse – therefore termed “gliapse” by the authors (Barcia et al., 2012). Importantly, application of Fasudil completely prevented microglial activation and fully rescued TH+-cell numbers (Barcia et al., 2012). However, it is still possible that the *in vivo* rescue of dopaminergic neurons is at least in part neuron-intrinsically mediated by non-canonical anti-apoptotic pathways such as Akt or ERK. Supporting this, an AAV2-mediated knock-down of ROCK2, preferentially expressed in neurons, induced ERK-activity *in vitro*, partially rescued the number of TH+-cells and mildly improved the motor-phenotype *in vivo* (Saal et al., 2015). A cell-type-specific ROCK-inhibition in microglia or neurons would therefore be an interesting approach to further clarify the detrimental contribution of microglia versus neuron-intrinsic ROCK-activity in PD.

## Multiple sclerosis (MS)

MS is a clinically diverse chronic CNS-disease characterized by inflammatory cell plaques in the white matter, loss of myelin and oligodendrocytes as well as gliosis (reviewed inGoldenberg, 2012; Duffy et al., 2014). The etiology of MS is largely unknown. However, together with histopathological findings, genetic risk factors such as variations of the major histocompatibility complex (MHC) (International Multiple Sclerosis Genetics et al., 2011) argue for an important role of neuroinflammation. Blood brain barrier (BBB) breakdown with infiltrating peripheral immune-cells is a characteristic event in the natural history of MS (reviewed in Ortiz et al., 2014). Interestingly, microglia are activated in advance of peripheral immune cell infiltration, underlining the importance of these cells in MS-disease progression (Ponomarev et al., 2005). Alternatively, it was suggested that MS is in fact a degenerative disorder (reviewed in Stys et al., 2012) which is supported by axonal damage not exclusively occurring in areas affected by auto-immunity (Trapp et al., 1998). At least, those axonal transections point toward a neurodegenerative component in MS-pathology.

Experimental autoimmune encephalomyelitis (EAE) has been extensively employed as an MS-animal model. Auto-immunization with myelin-sheet protein components such as myelin, oligodendrocyte glycoprotein (MOG), or Proteolipid Protein (PLP) induces pathological features similar to MS including neuroinflammation, demyelination, and neuronal damage (reviewed in Baxter, 2007). Importantly, ROCK upstream effector RhoA was upregulated in MS-patient plaques (Tajouri et al., 2005), and a PLP-induced EAE-mouse model exhibited an enhanced ROCK-activity in the CNS (Sun et al., 2006). Consistently, Fasudil application either in a preventive or therapeutic paradigm reduced clinical severity in different EAE-models (Sun et al., 2006; Hou et al., 2012). This was accompanied by a reduction of infiltrating inflammatory cells, demyelinated areas, and axonal transactions. Moreover, immune cells isolated from Fasudil-treated mice exhibited a decreased potential for immune response against EAE-inducing antigen, indicating a major role of ROCK-activity in mediation of auto-immunity by inflammatory cells (Sun et al., 2006). However, a chronic disease like MS demands long-term treatments with a minimal amount of side-effects. Thus, alternative application routes combining high CNS-availability with low peripheral abundance are favorable. Interesting results in this context were obtained by intranasal delivery of a Fasudil-derivate in a MOG-induced EAE mouse model (Li et al., 2014b). This treatment-regimen efficiently reduced demyelination, improved clinical scores, and enhanced body weight. Besides a reduction of neuroinflammation, the authors reported elevated BDNF- and NGF-levels suggesting an involvement of neurotrophic pathways by a yet unknown mechanism (Li et al., 2014b). However, this co-occurrence of neurotrophic factor-expression and ROCK-inhibition suggests an induction of pro-survival signals by non-canonical ROCK-crosstalk. Therefore, it is possible that beneficial effects of ROCK-inhibition in EAE are not only based on inhibition of neuroinflammation but are also mediated neuron-intrinsically.

## Alzheimers disease (AD)

The central clinical manifestations of AD are progressive cognitive deficits, namely memory, and learning disabilities. The neuropathological picture includes hippocampal atrophy with neuronal loss and reduced synaptic contacts. However, the characteristic histological findings defining AD are extracellular senile plaques and intraneuronal neurofibrillary tangles (reviewed in Perl, 2010). While the first is composed of amyloid-β (Aβ) peptides, the latter consists of hyper-phosphorylated tau-protein (reviewed in Bloom, 2014). The causal nature of Aβ-pathology is still under debate and findings of Aβ-accumulations without clinical manifestations question a strict relationship between Aβ-plaques formation and AD-symptoms for late-onset sporadic AD (reviewed in Moreno-Trevino et al., 2015). In contrast, mutations in the amyloid precursor protein (APP) as well as in presenilin 1 and 2 (PSEN1, 2) which participate in APP-processing cause hereditary early onset AD (Selkoe, 2001; Haass et al., 2012). This hints for an etiology based on Aβ-synthesis and secretion pathways. Thus, idiopathic and familial AD might differ in etiology, however, idiopathic AD-brains display enhanced neuronal ROCK2-protein levels (Herskowitz et al., 2013) as well as an altered subcellular distribution of its upstream effector RhoA which is a characteristic in common with genetic AD-mouse models (Huesa et al., 2010). Moreover, several studies demonstrated a role of the ROCK-pathway in Aβ-synthesis and secretion (Zhou et al., 2003; Herskowitz et al., 2013). Thus, the ROCK-pathway is a common target for both, idiopathic and familiar AD.

Intracerebroventricular Aβ-injections in rats lead to severe cognitive impairments, neuronal damage and an inflammatory response (Song et al., 2013) and can be employed as an AD-model reflecting Aβ-downstream effects. Interestingly, Fasudil-application rescued spatial learning and memory deficits as well as apoptosis phenotype in the AD-rat hippocampus. Moreover, inflammatory response indicated by enhanced IL-1β, TNFα, and NFκB-production was reduced back to normal levels (Song et al., 2013). Those effects might be mechanistically attributable to an inhibition of neuroinflammation or to an induction of anti-apoptotic neurotrophic pathways via non-canonical ROCK-crosstalk partners; however, the latter has not been tested in this AD-model yet.

Despite its action on neuroinflammation, several studies revealed a direct molecular role of the ROCK pathway within the Aβ-biosynthesis pathway. Plasma membrane bound APP is subjected to the non-amyloidogenic pathway by α-secretase cleavage leading to non-toxic sAPPα. When internalized, APP can be subsequently cleaved by β-secretase BACE1 and γ-secretase leading to toxic Aβ-production of different peptide lengths (reviewed in Bu, 2009). Importantly, Y27632-mediated ROCK inhibition changes γ-secretase cleavage specificity thereby shifting toxic Aβ_1−42_-peptide production to Aβ_1−38_ (Zhou et al., 2003), which is less toxic and might even be protective in combination with Aβ_1−42_ (Vandersteen et al., 2012). However, total Aβ-production was not changed. Interestingly, ROCK-isoforms differentially act on the amyloidogenic pathway: While ROCK1 knock-down enhances total Aβ-production *in vitro*, ROCK2 inhibition by the isoform-specific compound SR3677 reduced total Aβ-levels *in vitro* and *in vivo* (Herskowitz et al., 2013). Mechanistically, the authors suggested a regulation of BACE1-activity by ROCK2-mediated phosphorylation in combination with ROCK2-mediated APP phosphorylation triggering its processing (Herskowitz et al., 2013). In summary, ROCK-inhibition demonstrated efficacy in an AD-rat model most probably by inhibiting neuroinflammation. However, ROCK2-specific inhibition might be even more promising specifically targeting amyloidogenic pathways. In this context, ROCK1-inibition even might have a detrimental effect by enhancing Aβ levels. Thus, beneficial effects of global ROCK inhibition reducing neuroinflammation but enhancing Aβ biosynthesis via ROCK1 have to be trade-off against a specific ROCK2-inhibition without targeting glial cells.

## Huntington's disease (HD)

HD is a hereditary neurodegenerative disease caused by CAG-repeats within the huntingtin (Htt)-gene (The Huntington's Disease Collaborative Research Group, 1993). Thereby, the number of CAG-repeats correlates with the clinical onset which typically manifests by involuntary movements, and increasing motor- and cognitive dysfunctions. Consistent with the clinical picture, neuronal subpopulations display a selective vulnerability in HD with atrophy and neuronal degeneration in basal ganglia and cortex (reviewed in Shoulson and Young, 2011). The exact mechanism of neurodegeneration still remains unknown. However, mutant huntingtin aggregates or oligomers within neurons might play a crucial role (Legleiter et al., 2010).

Interestingly, the ROCK-pathway is directly involved in mutant huntingtin degradation and aggregate formation: Y27632 enhanced Htt-degradation thereby reducing its aggregation *in vitro* (Bauer et al., 2009), possibly by proteasome-activation and macroautophagy (Bauer and Nukina, 2009). Moreover, Htt aggregation is facilitated by ROCK-dependent phosphorylation of vimentin which finally leads to enhanced levels of free IP3R1. As IP3R1 facilitates mutant htt-aggregation, the authors proposed a model where ROCK-inhibition inhibits htt-aggregation via vimentin-IP3R1 axis (Bauer et al., 2011, 2012). Alternatively, a mechanism based on a direct molecular interaction of Htt was proposed: The ROCK-downstream target profilin-1, an actin binding protein and a major regulator of actin-dynamics, as well as the neuronal isoform profilin-2a, directly interact with huntingtin (Goehler et al., 2004; Shao et al., 2008). This interaction prevented Htt-aggregation indicating a beneficial effect in HD. Importantly, ROCK-mediated phosphorylation of profilin at Serine-137 inhibited its aggregation suppressing activity, suggesting beneficial effects of ROCK-inhibition in HD mediated by profilin-dephosphorylation (Shao et al., 2008). Consistently, Y27632 partially rescued photoreceptor neurons in a HD-*Drosophila* model (Pollitt et al., 2003). However, ambiguous results were obtained in R6/2 HD-model mice. Y27632 only mildly improved motor-performance, but had no effect on lifespan, cellular atrophy or aggregate formation (Li et al., 2009b). This lack of effects might be due to an insufficient dosing-regimen as there was no impact on profilin-phosphorylation (Li et al., 2009b). Intravitreal Fasudil application in the same model reduced retinal phospho-profilin1 staining and partially rescued retinal dysfunction (Li et al., 2013). Although retinal dysfunction might not be a feature of HD (Petrasch-Parwez et al., 2005), ROCK inhibition therefore proofed its beneficial potential on the functional level. However, mechanisms underlying those effects remain unclear as Htt-aggregation state, neuroinflammation, neuronal survival, or axonal regeneration have not been measured in a ROCK-inhibited HD-mouse model yet. Interestingly, neuronal survival and axonal regeneration in a primary HD *in vitro*-model could both be rescued by ROCK-inhibition (Deyts et al., 2009) suggesting the inclusion of such outcomes in future *in vivo* studies.

## Spinal muscular atrophy (SMA)

SMA is a monogenic motoneuron-disease affecting lower motoneurons progressively leading to paralysis, muscle atrophy and –in severe cases– to death. SMA is caused by deletions or loss-of-function mutations of the survival of motoneuron 1 (*Smn1*)-gene (Lefebvre et al., 1995). Humans harbor a second gene, *Smn2*, coding for the same protein. However, only low amounts of functional full-length protein is expressed from *Smn2* (Lorson et al., 1999). While complete SMN-loss is embryonically lethal, low SMN-levels lead to SMA and consistently, the number of *Smn2*-gene copies inversely correlates with disease severity (Taylor et al., 1998). The SMN-protein is ubiquitously expressed, and not surprisingly severe SMA with low SMN levels is considered not to be of pure motoneuron-pathology rather than being a multi-system disorder (Shababi et al., 2014). However, motoneurons are still preferentially affected, arguing in favor of focusing on pathomechanisms specific for these cell-types. The role of ROCK in SMA in different cellular contexts has been extensively studied and already excellently reviewed (Coque et al., 2014), so that we will address this only at a glance.

Importantly, ROCK-activity is enhanced in SMA-mouse model spinal cords and inhibition of ROCK by Y27632 or Fasudil significantly enhanced survival, rescued neuromuscular junction (NMJ) morphology, and muscle atrophy (Bowerman et al., 2010, 2012). Consistently, neuronal SMA-*in vitro* models displayed an hyper-active ROCK-signaling axis, indicating a neuron-intrinsic mechanism (Bowerman et al., 2007; Nolle et al., 2011; Hensel et al., 2014). Based on a direct interaction of the SMN-protein with the ROCK-downstream target profilin2a, the neuronal profilin-isoform, we proposed a molecular mechanism of neurotoxicity induced by SMN-depletion (Nolle et al., 2011): SMN-reduction leads to changed profilin2a-homeostasis and an enhanced profilin2a-ROCK binding. This finally results in a hyper-phosphorylation of profilin2a and a sequestration of ROCK from other downstream-targets such as cofilin or myosin light chain phosphatase (MLCP). Accordingly, those ROCK-targets were hypo-phosphorylated and actin-composition changed towards more stable, less dynamic, filamentous (F) actin (Nolle et al., 2011). This might have important implications for neuronal motility processes based on actomyosin contractility. Accordingly, neurite outgrowth defects were reported in SMA *in vitro* models (Van Bergeijk et al., 2006; Nolle et al., 2011). However, involvement of other actin-based motility processes such as axonal transport or synaptic vesicle cycling is possible. In this model, beneficial effects of ROCK-inhibition are mainly mediated by reduced phosphorylation of neuronal profilin, however, positive effects on gliosis which is a well-known feature of SMA (Simic, 2008) is likely possible.

Besides an up-regulated ROCK-pathway, also the ERK-signaling axis is hyper-activated in SMA model mice spinal cords (Branchu et al., 2013). In contrast to the classical view of ERK as an anti-apoptotic, neurotrophic pathway this over-activation acts detrimental on SMA-phenotype as ERK-inhibition by the small inhibitor Selumetinib enhanced survival of SMA-mice (Branchu et al., 2013). Therefore, the authors suggested an SMA-specific mechanism based on elevated SMN-expression. According to the *in vivo* situation, we could confirm ERK hyper-activation in motoneuron-like cells *in vitro* (Hensel et al., 2012, 2014). Moreover, ROCK and ERK are bi-directionally linked, as ROCK inhibits ERK and *vice versa* (Hensel et al., 2014). This crosstalk was observed in a timeframe of minutes making it unlikely that expressional changes represent the underlying mechanism. Interestingly, the crosstalk became uni-directionalized in SMA-model cells, as ERK was not capable to down-regulate ROCK selectively in SMA-cells. In summary, this leads to a co-hyperactivation of the ROCK- and ERK-signaling axes in SMA-cells (Hensel et al., 2014). As both pathways are potential therapeutic targets, we suggested a ROCK, ERK co-inhibition using a combination of Fasudil and Selumetinib. As detrimental ERK-hyper-activation could potentially be enhanced by ROCK-inhibition via ROCK-ERK crosstalk, we even expect synergistic effects of a combined treatment approach (Hensel et al., 2014).

Another crosstalk partner of ROCK is the phosphatase and tensin homologue deleted on chromosome ten (PTEN). PTEN mainly antagonizes the phosphatidylinositol 3 kinase (PI3K) pathway by hydrolyzing the second messenger phosphatidylinositol (3,4,5)-triphosphate (PIP3) thereby blocking the recruitment and activation of Akt (reviewed in Song et al., 2012; Hopkins and Parsons, 2014). It was shown that ROCK directly interacts and phosphorylates PTEN at several sites (Li et al., 2005; Hopkins et al., 2014). In non-neuronal cells, activated ROCK1 induced PTEN-activity resulting in an inactivation of Akt (Li et al., 2014a). Interestingly, *in vitro* data revealed that ROCK2, the neuronal ROCK-isoform, more potently phosphorylates PTEN than ROCK1 (Song et al., 2009). Thus, neuron-intrinsic ROCK-Akt crosstalk might be mediated via PTEN and enhanced ROCK-activities in SMA might lead to reduced Akt-activation via PTEN. However, while reduced Akt-activation has been pre-symptomatically observed in SMA-mice spinal cords (Branchu et al., 2013), to our knowledge, PTEN activity has not been monitored in SMA models so far. Importantly, increasing evidence has occurred about the role of PTEN in SMA: Knock-down of PTEN in cultured motoneurons as well as in SMA mice increases activity of the Akt-pathway, decreases disease severity and moderately enhances survival of SMA mice (Ning et al., 2010; Little et al., 2015).

## Amyotrophic lateral sclerosis (ALS)

ALS is a motoneuron disease not only affecting lower motoneurons in spinal cord but also leading to upper motoneuron and brainstem degeneration. Clinical symptoms include muscle weakness and paralysis leading to death 3–5 years after onset, which typically develops in advanced adulthood (reviewed in Chen et al., 2013b). About 90% of cases are sporadic with an unknown etiology; however, several risk genes with different penetrance have already been identified (Chen et al., 2013b). Mutant Superoxide dismutase 1 (SOD1) was the first gene identified to be involved in familiar ALS (Rosen et al., 1993), and the high penetrance allele SOD1^G93A^ (Synofzik et al., 2010) has been employed to generate the most extensively used ALS-mouse model (Gurney, 1997).

Interestingly, SOD1^G93A^-mice exhibited an enhanced ROCK-activity, elevated PTEN-activity while the Akt-pathway was down-regulated. Moreover, ROCK-inhibition by Fasudil-treatment completely rescued PTEN/Akt activity (Takata et al., 2013). This was accompanied by delayed symptom onset, enhanced survival and a partial rescue of motoneuron-numbers within spinal cord of SOD1^G93A^-mice (Takata et al., 2013). Interestingly, Fasudil-application in another study not only enhanced survival but also motor functions in SOD1^G93A^-mice. Moreover, motoneuron degeneration and nerve fibers were partially rescued (Tonges et al., 2014a). While the numbers of astroglial cells was reduced, Fasudil-treatment further enhanced microglia cell numbers but shifted morphology to the beneficial M2-microglia mechanism (Tonges et al., 2014a), which is in accordance with *in vitro* data (Zhang et al., 2013). Thus, two different studies suggested different mechanisms underlying beneficial effects of ROCK-inhibition in ALS-mouse models: (i) Altering the microglia-subtype composition, or (ii) inducing anti-apoptotic signaling via non-canonical ROCK-crosstalk. However, also neuron-intrinsic canonical ROCK-signaling, controlling axonal regeneration, is a candidate-mechanism. Involvement of ROCK-pathway and actin-dynamics in ALS-pathogenesis has recently been supported by newly identified familial ALS-genes: Mutations in the profilin1-gene are associated with high risks to develop ALS (Wu et al., 2012). Among those, profilin1^T109M^ represents a phospho-site mutation (Ingre et al., 2013) indicating an important role of profilin-phosphorylation However, it is unclear whether ROCK is an upstream-kinase for this site.

## Summary and future perspectives

Taken together, ROCK-inhibition ameliorates clinically relevant outcomes in rodent models of several NDDs making it a promising future treatment-strategy. However, with regard to chronic diseases detrimental long-term effects have to be considered highlighting alternative approaches such as non-systemic application routes as well as ROCK-isoform specific inhibition. With regard to the underlying mechanisms, neurons as well as neuroinflammatory cells such as microglia seem to be involved in mediating the beneficial effects of ROCK-inhibition. Genetic models with cell specific ROCK-depletion would greatly improve our understanding of the exact contribution by different cell-types. Moreover, little is known about the ROCK-dependent activation-mechanisms of neuroinflammatory cells on the molecular level. While ROCK-crosstalk with neurotrophic pathways is a relevant mechanism in neurons, such mechanisms have not been demonstrated in microglia yet. At this point, it has to been considered that elucidiation and analyses of single pathways and crosstalking molecules may limit our view on regulatory mechanisms. Instead, future work has to determine important nodes and edges as basic components of a molecular network with ROCK at its center. For example, network analyses could reveal new feedback loops and other network motifs relevant to determine direct and indirect effects of inhibitory drugs. Such a network should include multi-dimensional data on expression as well as on activation states to truly understand the flow of information in physiological and diseased conditions.

### Conflict of interest statement

The authors declare that the research was conducted in the absence of any commercial or financial relationships that could be construed as a potential conflict of interest.
